# Intimate Partner Violence among Colombian Population: Exploring Various Types of Abuse

**DOI:** 10.21500/20112084.7402

**Published:** 2025-10-21

**Authors:** Javier Andrés Gómez-Díaz, Leonardo Barón-Birchenall

**Affiliations:** 1 Corporación Universitaria Minuto de Dios - UNIMINUTO, Bogotá, Colombia Corporación Universitaria Minuto de Dios Corporación Universitaria Minuto de Dios - UNIMINUTO Bogotá Colombia

**Keywords:** Cyber abuse, partner abuse, sexual coercion, sexual devaluation, substance abuse, Ciberabuso, maltrato de pareja, coerción sexual, desvaloración sexual, abuso de sustancias

## Abstract

This study explored patterns of intimate partner violence (IPV) among the Colombian population, focusing on six abuse modalities-physical, psychological, sexual abuse due to devaluation, sexual coercion, and the lesser-studied economic and cyber abuse-along with their degree of mutuality and their associations with sociodemographic and behavioral factors including gender, sexual orientation, relationship status, age, Scholar level, and substance abuse. A cross-sectional correlational design was employed, involving 645 participants (non-probabilistic sample) who completed the Partners Abuse Scale. Compared to their counterparts, men, undergraduate students, alcohol-only consumers, and participants without an intimate partner during the study reported higher levels of physical abuse. Additionally, younger individuals were more likely to experience economic and cyber abuse. A general tendency toward mutual abuse across all types of violence was also observed. Overall, this research highlights IPV as a pressing public health concern and emphasizes the need for comprehensive preventive strategies targeting its various forms.

## Resumen

Este estudio exploró la violencia íntima de pareja (VIP) en población colombiana, abarcando seis modalidades de abuso: físico, psicológico, abuso sexual por desvaloración, coerción sexual, y las menos estudiadas abuso económico y ciberabuso. Se investigó el grado de mutualidad de los tipos de abuso y su relación con factores sociodemográficos y conductuales incluyendo sexo, orientación sexual, estado de la relación íntima, edad, nivel de educación, y abuso de sustancias. Mediante un diseño correlacional transversal se evaluó a 645 personas (muestra no probabilística) con la Escala de Abuso de Pareja. Los hombres, los estudiantes universitarios, los consumidores exclusivos de alcohol y quienes no tenían una pareja íntima durante el estudio informaron niveles más altos de abuso físico. Adicionalmente, se observó la mutualidad en todas las modalidades de abuso y la tendencia de los individuos más jóvenes a experimentar más violencia económica y ciberabuso. La investigación subraya la VIP como un problema de salud pública crítico, destacando la necesidad de estrategias preventivas integrales.

## 1. Introduction

Relationships involve a close emotional bond developed over time within specific contexts. However, in some cases, violence may occur between partners. This form of violence, known as *intimate partner violence* (IPV), encompasses various abusive behaviors perpetrated by a current or former partner, including physical, sexual, and psychological aggression (Alexander & Johnson, 2023; Ali et al., 2016; Tanskanen & Kivivuori, 2021). In this context, an intimate partner is defined as someone with whom an individual typically shares a close personal relationship, which may be characterized by emotional connectedness, regular contact, ongoing physical or sexual interaction, a shared identity as a couple, and mutual familiarity with each other's lives (Breiding et al., 2015).

Individuals who experience mistreatment from their partners often exhibit low self-esteem, struggle with adaptation, and experience psychological distress. They may also face additional challenges such as alco hol or drug abuse, anxiety, depression, or post-traumatic stress, which can, in turn, escalate into violent retaliation against the partner (Alqurashi et al., 2023; Control Disease Center, 2024; Ocampo, 2015).

Although manifestations of IPV tend to be underreported in both men and women (Juarros et al., 2024; Muzingili et al., 2024; Walsh & Stephenson, 2023), the World Health Organization (2024) reports that more than 25% of women aged 15 to 49 who have been in a relationship have experienced physical and/or sexual violence from their intimate partner at least once in their life-time-amounting to 476.4 million victims worldwide. This data supports classifying the phenomenon as a "shadow pandemic" (UN Women, 2020).

In the United States, 1 in 4 women and 1 in 9 men experience severe intimate partner physical violence, intimate partner sexual violence, and/or intimate partner stalking, leading to significant impacts on their daily lives, such as injuries, fearfulness, and post-traumatic stress disorder (National Coalition Against Domestic Violence, 2024). Similarly, Colombia-like other Latin American and Caribbean countries- has high rates of IPV, with evidence showing that both women and men have frequently been victims of violence by their partners or ex-partners (Lennon et al., 2021; Prado et al., 2024; Ripoll & Jaramillo, 2021).

Systematic reviews on Latin America conducted between 2000 and 2016 indicate that both Colombia and Peru have the highest number of cases of physical IPV against women, while Haiti reports the highest number of cases of sexual IPV against women (Bott et al., 2021). More recently, Moreno et al. (2024) conducted a reanalysis of the data based on scientific publications on IPV from 2020 to 2021, finding that, during this period, difficulties such as ineffetive communication, male violent behavior (intimidation and excessive control), economic problems, and disagreements regarding traditional gender roles have intensified.

Other authors have also raised concerns about the high levels of physical and se xual IPV against women in the following Latin American countries, listed here in order of highest to lowest prevalence: Bolivia, Ecuador, Costa Rica, and Colombia (Arango & Rubiano, 2019). In this regard, authors such as Weitzman et al. (2024) argue that various macrosocial patterns, particularly economic and political ones, directly influence IPV in the region. For instance, in conflict-affected countries such as Co lombia, the general population may perceive that breaking the rules is more common than following them, particularly in cases where wealthy individuals are involved in organized crime. This, in turn, undermines the credibility of the judicial system and fosters impunity for various harmful behaviors, including IPV. At the same time, micro-level interpersonal factors, such as direct exposure to violence, social, economic, and educational marginalization, and traditional beliefs about male dominance, contribute to the occurrence of IPV.

### 1.1 Intimate Partner Violence Modalities

There are different types of IPV, including *physical abuse*, which involves injurious behaviors and often appears to be a consequence of psychological abu se (Fitz Patrick et al., 2024; UN Women, 2020). Recurrent physical abuse involves actions such as pushing, grabbing, spitting, asphyxiating, or hitting one's partner (Viejo et al., 2018). It is important to note that some couples may consensually engage in inten se physical and verbal interactions to experience, generate, or enhance plea- sure, following mutually accepted ru les-a practice referred to as BDSM *(bondage and discipline, dominance and submission, sadism and masochism)*. In such cases, neither partner perceives them- selves as mistreated or abused, despite the similarities between BDSM practices and IPV (Brewer et al., 2023).

*Economic abuse* involves one partner con- trolling the other's finances, whether by taking money, preventing them from working, incurring debt in their name, and so forth (Mellar et al., 2024). It serves as a means of exerting power over the victim and promoting dependence (Newiak et al., 2024). In this regard, Klugman et al., (2024) found that most victims of domestic abuse also experience economic abuse, which can lead to psychological and workplace diffculties.

*Psychological abuse* involves the use of both verbal and non-verbal communication with the intention of causing mental or emotional harm to a partner and/or exerting control over them (Centers for Disease Control and Prevention, 2022). Unfortunately, this issue often goes unnoticed and may even be normalized within relationships due to cul tural and social norms, desensitization, and structural factors (Fernández et al., 2024; Wessells & Kostelny, 2022). Furthermore, elevated levels of psychological violence can escalate into physical violence (Charlot et al., 2025), often driven by factors such as jealousy, insecurity, and poor communication. These behaviors also serve as warning signs of physical abuse.

*Cyber abuse*, in turn, involves verbal and psychological mistreatment through so cial networks and information technologies (Cava & Buelga, 2018; Rogers et al., 2023). This type of abuse, which has been on the rise partly due to the increasing use of vir tual platforms, includes sending insulting messages, invading privacy by inspecting electronic communications without consent, promoting harm to others online, and exerting control through partner surveillance (Piquer et al., 2017; Bailey et al., 2024).

Regarding sexual abuse, it consists in forcing or attempting to force someone to take part in a sex act, sexual touching, or even a non-physical sexual event such as sexting, without consent (Centers for Disease Control and Prevention, 2022). For the purposes of this study, we distinguish between *sexual abuse due to devaluation* and *sexual coercion*. Sexual abuse due to devaluation is a form of emotional abuse where one person belittles or undermines another to manipulate them into engaging in sexual activity. This behavior may involve making demeaning comments about a partner's body or sexuality, humiliating or insulting them, or promoting feelings of insecurity or dependence. On the other hand, sexual coercion involves using threats, intimidation, or pressure to compel someone to engage in sexual activity against their will. This can include threats of violence or harm to oneself, the victim, or a loved one, as well as threats of withholding affection or approval, and inducing feelings of guilt or obligation.

The different types of IPV, and their interrelationships, can be conceptualized and understood in various ways. For example, various theoretical frameworks on IPV (Burelomova et al., 2018; Wood, 2015; Zavala & Kurtz, 2021) identify different forms of violence, often highlighting at least three interconnected types: *interpersonal, structural*, and *cultural*. Interpersonal violence is usually associated with visible aspects, such as physical violence, which, in the case of IPV, can also be sexual, psychological, or emotional, manifesting through deprivation or neglect, which are sometimes less visible. In contrast, structural and cultural violence are often indirect or institutionalized. There is usually no single perpetrator, and it may even be impractical to identify one.

Structural violence arises from the unequal distribution of power and resources within the social structure in which the individual develops. Cultural violence, on the other hand, involves the legitimizing character of both interpersonal (direct) and structural (indirect) violence, as seen in ca ses where a person is revictimized when reporting their case, leading to further harm to their mental health.

### 1.2 The Present Study

In this study, we explore the aforementioned six modalities of IPV among Colombian population, including their degree of mutuality (i.e., the extent to which both partners engage in abusive behaviors towards each other). To contextualize the phenomenon, we examine whether variations in the forms of IPV are associated with sociodemographic and behavioral fac- tors, including gender and sexual orientation, relationship status, age and scholar level, and substance abuse.

## 2. Method

### 2.1 Design and Participants

A cross-sectional correlational design was employed with a non-probabilistic sample of 645 participants (246 women and 399 men), with an average age of 25.3 years for men and 26.4 years for women, both cases having a standard deviation of 8.5 years. Participants were residing in Bogotá at the time of the study and were required to have either been in a current or past romantic relationship to participate.

Those who were in a relationship at the time of the study had, on average, been in their current relationship for 5 years and 3 months, while participants who were single during the study had previously been in relationships with an average duration of 2 years and 3 months. Additional sociodemographic information can be found in Table 1.

**Table 1:** Sociodemographic Information.

Variable	F	%
Gender		
Women	246	38.1%
Men	399	61.9%
Sexual Orientation		
Heterosexual	610	94.6%
Other	34	5.3%
Missing Data	1	0.2%
Couple Status		
Windowed	61	9.5%
Single	243	37.7%
Divorced	103	16.0%
Married	218	33.8%
Non-Formal Relationship	19	2.9%
Missing Data	1	0.2%
Scholar Level		
Basic	144	22.3%
Undergraduate	454	70.4%
Postgraduate	44	6.8%
Missing Data	3	0.5%
Substance Abuse		
Alcohol (only)	375	58.1%
Alcohol and Another Substance	38	5.9%
None	229	35.5%
Missing Data	3	0.5%

### 2.2 Instrument

We employed the *Partners Abuse Scale* (PAS; Barajas et al., 2021). This psychometric test utilizes self-directed statements about IPV to assess the degree to which participants have experienced victimization. The PAS employs a 4-point Likert scale, as follows: 1 = Never, 2 = At least once, 3 = More than once, 4 = Several ti mes. In this test, IPV is categorized into six abuse modalities: **1. Physical:** 14 items, Cronbach's Alpha =.92 (sample item: *I feel abused or mistreated when my partner has hurt me with a sharp or pointed object*); **2. Economic:** 20 items, Cronbach's Alpha =.92 (sample item: *I feel abused or mis- treated when my partner has handled my money without my consent*); **3. Psycho- logical:** 17 items, Cronbach's Alpha =.92 (sample item: *I feel abused or mistreated when my partner has made jokes at my expense that bother me*); **4. Cyber abuse:** 12 items, Cronbach's Alpha =.85 (sample item: *I feel abused or mistreated when my partner has posted private information about me on some electronic media*); **5. Sexual due to devaluation:** nine items, Cronbach's Alpha =.79 (sample item: *I feel abused or mistreated when my partner has destructively criticized my sexual behavior*); **6. Sexual coercion:** ten items, Cronbach's Alpha =.80 (sample item: *I feel abused or mistreated when my partner has prevented me from using family planning methods*). The total consistency of the scale was high (Cronbach's Alpha =.96).

To obtain the total score for each scale, the scores given by participants for each item were summed. An option was added to each question to indicate if the act of violence was mutual (i.e., experienced or done by both partners).

### 2.3 Procedure and Statistical Analysis

Given that the population is difficult to quantify, as the exact number of individuals affected by IPV is unknown, we aimed to reach a representative sample size of 655 participants, based on the formula n = Z^2^ * p * q / e2 (where Z = 2.56, p = q = 0.5, and e = 0.05), with the assumption that men and women had an equal chance of being included in the study. Nevertheless, the sample may not be fully representative of the entire population. Participants were asked to complete a sociodemographic varia bles questionnaire and to sign an informed consent that contained the purposes of the study and the request for voluntary and anonymous participation. Data were statistically processed using SPSS.23. Since the variables did not show a tendency to normality, analyses were performed using non-parametric tests. Effect sizes were calculated using rank biserial correlations where applicable.

### 2.4 Ethical Considerations

The study was reviewed and approved by the ethics committee of the Corporación Uni versitaria Minuto de Dios - UNIMINUTO. In the case of four women who requested to know the results of their test, informed consent was obtained, and a psychological support pathway was activated upon noticing that they were victims of IPV based on their questionnaire responses.

## 3. Results

### 3.1 Gender and Sexual Orientation

Men reported higher levels of experiencing physical, economic, psychological, and cyber abuse (see Table 2). No significant differences were found based on the participants' sexual orientation.

**Table 2:** Abuse Modalities by Gender.

Abuse Modalities	Gender	X^-^/SD	Mann- Whitney U test	Effect size
	Men	17.27/5.84		
Physical			36510**	0.20
	Women	15.84/4.29		
	Men	19.33/6.59		
Economic			35723**	0.22
	Women	17.66/5.15		
	Men	29.18/10.00		
Psychological			37857**	0.10
	Women	17.66/5.15		
	Men	18.54/6.29		
Cyber Abuse			36744**	0.17
	Women	16.91/5.37		
	Men	9.51/3.02		
Sexual due to Devaluation			44731	0.02
	Women	9.33/2.42		
	Men	14.53/4.34		
Sexual Coercion			41634	0.07
	Women	14.05/3.95		

*p < 0.05; **p < 0.01.

### 3.2 Relationship Status

Participants who were in a relationship be- fore the study but not during it reported higher levels of experiencing physical, psycho- logical, cyber abuse, and sexual abuse (both coercion and due to devaluation) compared to those who were in a relationship during the study (see Table 3).

**Table 3:** Abuse Modalities by Relationship Status.

Abuse Modalities	Relationships_-_status	X/SD	Mann-Whitney U test	Effect size
	Present	16.30/5.12		
Physical			38392*	0.10
	Previous	16.58/4.74		
	Present	18.19/5.85		
Economic			39509	0.08
	Previous	18.54/5.74		
	Present	27.36/9.49		
Psychological			31198**	0.21
	Previous	30.24/10.07		
	Present	17.03/5.67		
Cyber Abuse			34013**	0.18
	Previous	18.52/5.92		
	Present	9.22/2.67		
Sexual due to Devaluation			35725**	0.17
	Previous	9.77/2.62		
	Present	13.87/4.03		
Sexual Coercion			33602**	0.21
	Previous	14.97/4.18		

*p < 0.05; **p < 0.01.

Note: the relationship status 'Present' indicates that participants were in a relationship during the study, whereas 'Previous' refers to participants who were in a relationship before the study but not during it.

### 3.3 Age and Scholar Level

Differences were found in economic violence (Kruskal-Wallis[3] = 29.88; p < 0.01) and cyber abuse (Kruskal-Wallis[3]= 12.38; p = 0.01) according to the age group. In both cases, younger participants (up to 21 years old) reported higher scores than those in older age groups (Economic violence: Dunn's z = -4.26; p < 0.01; Cyber abuse: Dunn's z = 2.96; p < 0.01).

Based on the level of education, differences were only found in physical violence (Kruskal-Wallis = 6231; p = 0.04), with a small effect size (Eta2 = 0.01). Post-hoc tests (Dunn's test) revealed differences both between basic and postgraduate education, as well as between postgraduate and undergraduate education (see Table 4). The highest levels of experiencing physical violence were reported by the undergraduate group (x^-^ = 16.58, SD = 5.41), compared to the basic education group (x^-^ = 16.22, SD = 3.92) and the postgraduate group (x^-^ = 15.07, SD = 3.14). 

**Table 4:** Post-hoc Analyses of Physical Abuse by Scholar Level.

Scholar Leve (Physical Abuse)	Z	Wi	Wj	p-Holm
Basic-Undergraduate	0.658	322,022	311,275	0.511
Basic-Postgraduate	2.461	322,022	249,134	0.042*
Undergraduate-Postgraduate	2.291	311,275	249,134	0.044**

* p < .05 based on Holm's test.

### 3.4 Substance Abuse

Higher levels of experiencing economic (Mann-Whitney U = 39313.5; p = 0.003) and physical violence (Mann-Whitney U = 39154; p = 0.001) were reported by alcohol-only consumers. In both cases, with a small effect size (economic violence: Cohen's D = 0.137; physical violence: Cohen's D = 0.142). Similarly, higher levels of experiencing physical violence (Mann-Whitney U = 13072.5; p = 0.045) were reported by consumers of alcohol and another substance, again with a small effect size (Cohen's D = 0.18). See Table 5.

**Table 5:** Substance Abuse and Abuse Modalities.

Abuse	Abuse Modalities		X	SD
		yes	18.547	5.617
	Economic	no	17.955	6.082
Alcohol (only)		yes	16.717	5.277
	Physical	no	15.926	4.510
Alcohol and Another Substance	Physical	yes	16.974	3.767
		no	16.355	5.060

Note: Only significant results are shown.

### 3.5 Correlations

A positive correlation was identified among all forms of abuse, with the strongest associations observed between economic and psychological abuse and between economic and physical abuse. Additionally, a strong positive correlation was found between the two types of sexual abuse (coercion and devaluation; Table 6). 

**Table 6:** Correlations Between Abuse Modalities.

Modalities Abuse		PH	EC	PS	CA	SD
Economic (EC)	Pearson's r	0.718***	-			
	Effect size	0.041				
Psychological (PS)	Pearson's r	0.675***	0.72***	-		
	Effect size	0.042	0.042	-		
Cyber Abuse (CA)	Pearson's r	0.545***	0.59***	0.683***	-	
	Effect size	0.041	0.041	0.042	-	
Sexual due to Devaluation (SD)	Pearson's r	0.649***	0.63***	0.645***	0.497*** -	
	Effect size	0.041	0.041	0.042	0.041	-
Sexual Coercion (SC)	Pearson's r	0.652***	0.631***	0.617***	0.531***	0.705***
	Effect size	0.041	0.041	0.042	0.041	0.041

***p < 0.001. PH: Physical.

Note: Effect sizes estimated using Fisher's z.

The positive correlations across all abuse types suggest that women and men who experience one form of abuse are more likely to experience others, highlighting the cumulative and overlapping nature of IPV. The strongest associations occur between economic and psychological abuse and between economic and physical abuse (Table 6), which supports the central role of economic abuse as a co-occurring mechanism that may accompany or reinforce physical and emotional domination, potentially increasing victims' dependence and vulnerability. Furthermore, the two dimensions of sexual abuse (coercion and devaluation) are strongly correlated (Table 6), indicating that these behaviors frequently co-occur within a broader pattern of sexual domination and degradation. This strong link emphasizes the need to consider sexual violence not only in terms of forced acts (coercion) but also as a form of emotional and psychological dehumanization (devaluation).

### 3.6 Mutuality

An evident tendency towards mutuality was observed as participants indicated that they had executed most of the violent behaviors against their partner as well, except for attempting to steal from their partner (Mann-Whitney U = 1210; p < 0.01) or stealing from their partner (Mann-Whitney U = 2422; p < 0.01), as forms of eco- nomic violence, and hitting their partner for receiving a compliment (Mann-Whitney U = 1801.5; p < 0.01), as form of physical violence. In these three cases the tendency was unidirectionality.

## 4. Discussion

In line with reports such as those by Rey (2008) and Mellar et al. (2024), in our study men reported experiencing more economic violence than women, but also reported higher levels of physical and psychological violence, and cyber abuse. In this regard, while a significant body of literature highlights women as victims of violence (e.g., Viejo et al., 2018 and references therein), it is important to note that gender differences, particularly regarding physical violence, are multifaceted and, in some cases, not statistically significant (Organization for Economic Co-Operation and Development, 2023). As for this, it has been proposed that categorical differences between women and men tend to nullify due to the various predisposing variables leading each gender to resort to violence as a misguided means of conflict resolution (Ma et al., 2023). Therefore, it is recommended to reassess the interpretation given to IPV. For instance, following the recommendation of Bartholomew et al. (2015) to avoid including samples from only one gender or treating only one gender as victims can lead to a broader and deeper understanding of IPV manifestations. Furthermore, future research should explore whether the impact of IPV on men and women is similar in terms of the intensity and frequency of harm.

On the other hand, the fact that men reported experiencing more economic, psychological, and cyber abuse could be associated with gender roles, power dynamics, and societal expectations. For example, the higher rates of economic abuse reported by men might come from the traditional expectation that men should be the main earners in a household. If they rely on their partner for financial support or face barriers to accessing money, they may be more vulnerable to this type of abuse. Regarding psychological and cyber abuse, men might be more likely to recognize and report these forms of violence compared to physical abuse. This could be because physical abuse is often more strongly linked to women. Additionally, in relationships with power struggles, men may become targets of emotional manipulation, including the use of technology for monitoring, threats, or harassment.

With respect to the relationship sta tus, participants without a current intimate partner during the study reported higher levels of abuse across all modalities except economic. These findings suggest that these forms of abuse may have been perpetrated by ex-partners, which is consistent with research by Li (2023), highlighting their significance in relationship dissolution. Concerning this, it is possible that individuals who are not currently in a relationship may still be struggling with the aftermath of abuse experienced during previous partnerships, particularly if those abusive dynamics were not fully addressed. Additionally, the absence of a current partner may provide individuals with a clearer perspective on past mistreatment, leading them to acknowledge and report it more readily.

Our findings also indicate significant differences in experiences of economic violence and cyber abuse based on age, particularly highlighting a concerning trend among younger individuals (up to 21 years old). This finding may indicate that younger individuals are more vulnerable to these forms of abu se, possibly due to factors such as limited financial independence, less experience in navigating relationships, or greater exposure to digital interactions where cyber abu se can occur. On the other hand, the lower scores reported by older participants may not necessarily indicate a lower prevalence of economic violence and cyber abuse. Instead, they could suggest underreporting or differing definitions of abuse, particularly if older individuals are less likely to recognize or report certain behaviors as abusive.

Regarding Scholar level, although the undergraduate group reported experiencing the highest levels of physical violence, it is worrying to find that regardless of the level of education, the different types of violence occurred with a similar frequency. There could be several factors contributing to this pattern. One possibility is that issues beyond education, such as socio-economic status, cultural norms, or relationship dynamics, may play a more significant role in determining the frequency of violence, as has been argued by Weitzman et al. (2024). Additionally, it's important to consider that education alone may not necessarily protect individuals from experiencing violence, as other factors like communication skills, coping mechanisms, and access to support networks also influence the dynamics of interpersonal relationships.

Another way to understand the fact that physical violence was reported more frequently among undergraduate participants is to consider how this stage of life may shape relationship dynamics. Undergraduate years often coincide with a period of transition, in which individuals experience greater autonomy, explore new social roles, and may engage in more unstable or short-term romantic relationships. These factors may contribute to elevated emotional intensity and, in some ca ses, conflict. Additionally, younger individuals may have less experience recognizing unhealthy relationship patterns or accessing support resources, which could increase their vulnerability to violence. Thus, rather than viewing education as unrelated to violence, it may be that specific characteristics of this educational stage interact with other psychosocial factors, influencing the likelihood of experiencing IPV.

Furthermore, in our study, participants who consumed only alcohol reported experiencing more physical and economic violence compa red to non-consumers of alcohol. Conversely, those who consumed alcohol along with another substance reported experiencing more physical violence. These findings highlight the complex relationship between substance consumption and experiences of violence, suggesting that different patterns of substance consumption may be associated with varying risks of experiencing different types of violence. Understanding these patterns can help inform targeted interventions aimed at reducing the incidence of violence among individuals who consume substances.

Regarding the observed tendency towards mutual exertion of IPV, it is important to re- cognize that various personal characteristics may permeate couple relationships, shaping contextual interactions that can lead to different forms of violence (UN Women, 2024; Weitzman et al., 2024). Since mutuality in IPV appears to extend beyond a single type of abuse in daily interactions, it is possible that if one member of the couple perpetrates violence against the other, it may elicit a simultaneous response. Nonetheless, such response may not necessarily involve the same method or intensity of violence, but rather employ tactics aimed at causing harm to the partner in diverse ways.

For example, participants of our study who reported experiencing sexual abuse, whether by coercion or devaluation, also reported mutual aggression in such instances ('you harass me, I devalue you' or 'you diminish my sexual behavior, I reject you'). In this respect, it must be noted that violence in the sexual sphere can significantly hinder healthy relationship development, as the aggressor, regardless of gender, tends to dominate their partner, leading to discomfort, dissatisfaction, and/or rejection (Brockstedt et al., 2025; Stockman et al., 2024).

Moreover, mutual IPV is not always classified according to the specific type of abuse, but rather treated as a general phenomenon of violence. In this regard, the identification of mutual abuse within specific violence modalities enables a more detailed characterization of IPV, facilitating a nuanced analysis of causality among manifestations and the relationships between different ways of perpetrating abuse.

It is also important to recognize the role of socioeconomic and governmental factors underlying IPV, particularly the microsocial and macrosocial patterns that contribute to the underreporting of victims (both men and women), including firsthand exposure to violence, marginalization in social, economic and educational spheres, and deeply rooted beliefs in male dominance. In this respect, the judicial system in Colombia has been notably permissive and ineffective in guaranteeing fair and timely access to comprehensive civil rights protection.

As a drawback, while our study results appear to be fairly generalizable, it is important to note that we did not use a fully probabilistic sample, which limits the representativeness of our findings. Also, although all participants resided in Bogotá at the time of the study, and it is reasonable to assume that a significant portion of them were from other parts of the country, it is recommended to analyze a more controlled sample from various regions across Colombia for a comprehensive understanding of the dynamics of IPV in the country. Moreover, given the severity of the harm that can potentially result from each behavior included in the Partners Abuse Scale, it is advisable to study the effect that weighting this severity would have on the validity evidence of the instrument.

Additionally, although the normality test indicates that the data do not exhibit such a tendency, future studies could normalize the raw scores to compare the scales under unified criteria. It is also advisable for the test to be verified using the Rasch model, as has been done with similar instruments for measuring IPV (e.g. Ayu et al., 2023). It is also recommended to contrast IPV by comparing the results with instruments that measure relationship satisfaction, such as the Love Bank Inventory (Chalmers, 2022), or the Mindful Partnering Measure (Seiter et al., 2021).

Finally, to further explore the dynamics of mutual abuse within intimate partner relationships, subsequent research may focus on identifying risk and protective factors that may help detect subtle signs of abuse. By doing so, clinical and social interventions aimed at preventing abuse and promoting peaceful resolution of interpersonal conflicts can be more effectively implemented.

## 5. Conclusión

Although maltreatment within couples is a common occurrence, it often remains invisible due to its nuanced characteristics. This includes the fact that it may not always leave visible signs, as is the case with psychological and economic abuse. The findings of our study emphasize the need to implement preventive strategies in healthcare, legal institutions, schools, and other family and couple-related contexts to mitigate the recurrence of this problem. Our research also indicates that IPV is a public health problem affecting both men and women, particularly among younger individuals. In this scenario, it is vital to recognize and support all victims to ensure that the complexities of IPV are addressed. Besides, ignoring the age factor may lead to inadequate support for younger victims, who may experience different forms and dynamics of IPV compared to older individuals.
